# Multiple Osteolytic Lesions Causing Hypercalcemia: A Rare Presentation of Acute Lymphoblastic Leukemia

**DOI:** 10.1155/2017/2347810

**Published:** 2017-07-17

**Authors:** Khalid Mahmood, Muhammad Ubaid, Syeda Taliya Rizvi

**Affiliations:** ^1^Faculty of Medicine and Allied Health Sciences, Dow University of Health Sciences, Karachi, Pakistan; ^2^Civil Hospital Karachi, Dow University of Health Sciences, Karachi, Pakistan

## Abstract

Acute lymphoblastic leukemia is characterized by unchecked proliferation of malignant lymphoblasts which replaces the normal bone marrow culminating in anemia due to red blood cells inadequacy as well as in easy bruising/bleeding secondary to insufficient platelets production. Even the white blood cells which are produced excessively are immature and abnormal. ALL is the most common hematological malignancy in children. Most commonly, patients present with lymphadenopathy, recurrent infections, bleeding, fatigue, and bone pains. Bone pains, often particularly involving long bones, occur in about 21–38% of cases and are due to overcrowding of bone marrow with malignant cells. Vast majority of children with ALL have thrombocytopenia and/or anemia with a normal or mildly elevated white blood cells count with the presence of lymphoblasts on peripheral smear. About 50% of children present with bleeding while about 75% of patients have platelet count 100,000/microL. Visceromegaly is not uncommon but osteolytic lesions and hypercalcemia are rather uncommon. We present a 22-year-old gentleman with generalized fatigue and bone pains without visceromegaly. There was severe hypercalcemia with normal parathyroid levels but multiple osteolytic lesions. Peripheral smear showed anemia without blasts, whereas a bone marrow biopsy revealed > 30% blasts with interspersed CD 10 positive cells.

## 1. Case Report

A 22-year-old male presented with pain and generalized weakness particularly involving proximal muscles of upper and lower limbs since the last 3 months. The weakness was accompanied by difficulty in getting up from a sitting position, climbing stairs, and raising arms above head. For the last two months, patient's symptoms were accompanied by fever and increased volume and frequency of urine throughout the day but particularly prominent at night, requiring the patient to wake up multiple times during sleep. However, no dysuria, hematuria, or flank pain was reported. He did not have any comorbid illnesses and his family history was unremarkable. Patient was normotensive. He did not complain of heat or cold intolerance or palpitations. His surgical and past medical history was insignificant. He did not smoke, drink alcohol, or use illicit drug.

On physical examination, mild wasting and proximal weakness were observed in all four limbs. The rest of neuromuscular examination was normal involving normal reflexes and normal muscle tone and no upper motor neuron lesions were appreciable. No truncal obesity, striae, or skin changes were noticeable. Mild anemia was also observed, however, without any lymphadenopathy, hepatomegaly, or splenomegaly. Cardiovascular and pulmonary exam was normal.

### 1.1. Laboratory

Laboratory investigations showed hemoglobin 6.2 g/dL, platelet count 223000 cells/mcL, and TLC at 8600 cells/mcL. Peripheral smear showed normocytic, mild hypochromic anemia without any erythroblasts. Blood urea nitrogen was 27 mg/dL and creatinine was 1.3 while electrolytes were unremarkable. Albumin was found to be 2.9 g/dL and total proteins were 5.6 g/dL. His urine analysis was negative for proteins and liver function tests, and clotting profile was normal. ESR and CRP were 68 mm/hr and 245.3 mg/L, respectively. Lastly, serum calcium levels were 14.6 and serum phosphorus levels were normal at 5.0. Serum PTH was 5.0, with a normal vitamin D3. Urine analysis was insignificant as were urinary electrolytes.

### 1.2. Radiology

His chest X-ray was unremarkable, whereas X-ray pelvis revealed multiple osteolytic lesions in the iliac bones, and another X-ray also showed similar osteolytic lesions in the cranium. A bone scintigraphy revealed nonhomogenous increase in tracer uptake in the left hip joint as well as the left sacroiliac joint. A computerized tomographic contrast scanning of the chest and abdomen showed multifocal osteolytic lesions in axial skeleton.

### 1.3. Immunoelectrophoresis

Suspecting some form of paraproteinemias, subsequent investigations including immunoelectrophoresis and immunofixation of serum proteins were carried out which showed no monoclonal gammopathy.

### 1.4. Bone Marrow Studies

Finally, a bone marrow biopsy was done, which revealed marrow studded with 31% blasts, with myeloid precursors constituting 64% of the total. Immunohistochemistry revealed that the cells were positive for Tdt (terminal deoxynucleotidyl transferase), CD 10 (cluster differentiation), and CD 79a receptors and negative for CD 34. As a result, a diagnosis of precursor B-cell acute lymphoid leukemia was made.

The patient was treated with judicious use of intravenous fluids, intravenous furosemide, and a single dose intravenous ibandronate. He was referred to specialized hematology center of the city. He is in regular follow-ups there and also with our department.

## 2. Discussion

Acute lymphoblastic leukemia is the cancer of white blood cells and the bone marrow that preferentially involves the immature lymphocytes called lymphoblasts. ALL results in malignant proliferation of the lymphoblasts that replaces the normal hematopoietic cells of the bone marrow resulting in cytopenias and extramedullary hematopoiesis. ALL accounts for 15% of all adult leukemia but it is the most common hematological malignancy of childhood accounting for 30% of all malignancies of childhood. ALL has a bimodal age distribution with peak incidence at 2–5 years of age and a second peak above 50 years of age [1–3].

Hypercalcemia in conjunction with osteolytic bone lesions is a common feature of several diseases, including multiple myeloma and adult T-cell leukemia; however, it is rarely found in pediatric/adolescent ALL [2]. In a large retrospective study carried out at St. Jude's Children's Cancer Hospital over a period of 29 years and involving a group of more than 6000 pediatric malignancy patients, only 0.4% of patients being treated had hypercalcemia during the course of malignancy. In a subgroup of 2,816 of these patients who had acute leukemia/lymphoma, the prevalence of hypercalcemia at the time of diagnosis was found to be only 0.3%. In another study carried out, the incidence of hypercalcemia in a group of 83 patients with B-ALL subtype of ALL and the incidence of hypercalcemia were noted to be 4.8% [4], still accounting for a very small percentage of ALL population presenting with hypercalcemia; only a very small proportion of these patients actually have osteolytic lesions [1, 5]. So the presence of hypercalcemia with severe osteolytic lesions was something different to usual findings of ALL.

ALL patients presenting with hypercalcemia and osteolytic lesions are older (second decade of life), have a low or normal WBC count, and do not have significant blast cells in peripheral blood smear [6]. Presence of hypercalcemia and osteolytic lesions in ALL is unusual. However, when ALL does present with such features, many of the hypercalcemic patients are observed to be positive for immunophenotype CD 10 and negative for CD 19. Similar findings were present in our patient. The blast cells usually have a pre-B-ALL phenotype with widespread osteolytic lesions and hypercalcemia and so was the case in this patient.

Overall, the most prevalent cause of malignancy related hypercalcemia is ectopic production of a hormone called Parathyroid Hormone related Protein (PTHrP) which exerts similar effects to PTH (parathyroid hormone) including increased osteoclastic activity with resulting bone resorption, augmented reabsorption of calcium from kidneys, and increased renal phosphate excretion [2]. However, the exact cause of hypercalcemia in ALL has not been established well but there have been postulations outlining multiple mechanisms underlying hypercalcemia encountered in ALL. One of the postulations is that PTHrP associated hypercalcemia as found in other malignancies manifesting with hypercalcemia is the main pathway [1, 7]. Our hospital's hematology lab does not have this facility to identify blast producing Parathyroid Hormone related Protein (PTHrP); otherwise it would have further strengthened this. A study also suggests that a rise in levels of certain cytokines including IL-1, IL-6, TNF, PGE2, and TGF-alpha might be responsible for hypercalcemia in ALL [8]. The cytokines are believed to act as osteoclast-activating factors, resulting in the lytic bone lesions, osteopenia, and osteolytic lesions, mainly involving the axial skeleton.

Thus, presentation of an ALL patient with hypercalcemia and osteolytic lesions along with deceptively low WBC counts and no blast cells on peripheral smear, as in the case of our patient, is a very rare but relevant finding. Thus, while dealing with patients suspected of hematological malignancies, such rare presentation should not be overlooked. The prognosis of childhood ALL with osteolytic bone lesions and hypercalcemia is apparently not poor; however, this has not been established and requires further investigation [7].
